# Molecular chaperones and photoreceptor function

**DOI:** 10.1016/j.preteyeres.2008.03.001

**Published:** 2008-07

**Authors:** Maria Kosmaoglou, Nele Schwarz, John S. Bett, Michael E. Cheetham

**Affiliations:** Division of Molecular and Cellular Neuroscience, UCL Institute of Ophthalmology, 11–43 Bath Street, London EC1 V 9EL, UK

**Keywords:** Retina, Neurodegeneration, Retinal dystrophy, Molecular chaperone, Rhodopsin, RP2, AIPL1, Heat shock protein, Hsp

## Abstract

Molecular chaperones facilitate and regulate protein conformational change within cells. This encompasses many fundamental cellular processes: including the correct folding of nascent chains; protein transport and translocation; signal transduction and protein quality control. Chaperones are, therefore, important in several forms of human disease, including neurodegeneration. Within the retina, the highly specialized photoreceptor cell presents a fascinating paradigm to investigate the specialization of molecular chaperone function and reveals unique chaperone requirements essential to photoreceptor function. Mutations in several photoreceptor proteins lead to protein misfolding mediated neurodegeneration. The best characterized of these are mutations in the molecular light sensor, rhodopsin, which cause autosomal dominant retinitis pigmentosa. Rhodopsin biogenesis is likely to require chaperones, while rhodopsin misfolding involves molecular chaperones in quality control and the cellular response to protein aggregation. Furthermore, the specialization of components of the chaperone machinery to photoreceptor specific roles has been revealed by the identification of mutations in molecular chaperones that cause inherited retinal dysfunction and degeneration. These chaperones are involved in several important cellular pathways and further illuminate the essential and diverse roles of molecular chaperones.

## 1 Introduction

### 1.1 Molecular chaperone function

Molecular chaperones are facilitators and regulators of protein conformational change (Ellis and Hartl, 1999). Importantly, they do not provide information with regard to the final protein structure. Chaperones bind to and stabilize conformers of other proteins, and via cycles of regulated binding and release are able to facilitate the correct fate of their client protein and reduce protein aggregation (Dobson, 2004). Through this mechanism, molecular chaperones play an essential role in many cellular processes. They have a principal role in protein folding, where they are involved in the *de novo* synthesis of polypeptides (Hartl and Hayer-Hartl, 2002), transport across membranes (Rapoport, 2007) and the refolding of proteins denatured by environmental stress (Nollen and Morimoto, 2002). The ability to respond to cellular stress is reflected by many molecular chaperones also being stress response proteins and bearing the name ‘heat shock protein’ (or Hsp) followed by an approximate molecular weight (e.g. Hsp70). Molecular chaperones also function in the oligomeric assembly and disassembly of protein complexes, controlled switching between active and inactive conformations of client proteins, intracellular transport and protein quality control and degradation (Ellis, 2006).

**Fig. 1:**
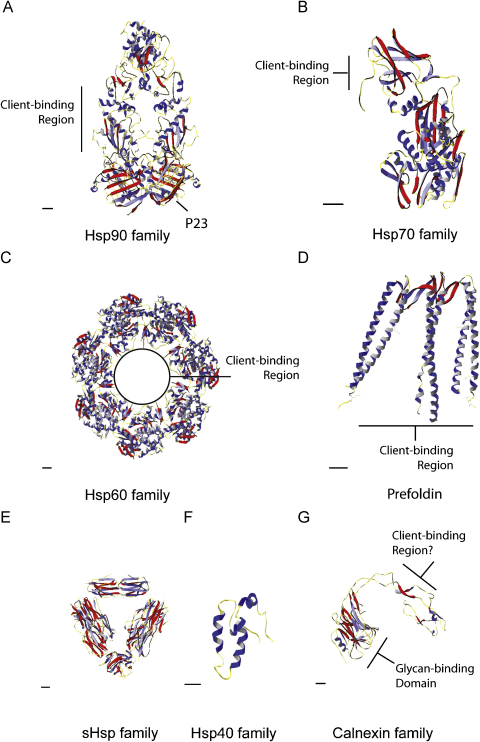
Different structural solutions to client protein binding in the major families of molecular chaperones. Ribbon diagrams showing representations of the structures and client protein binding sites of the following chaperones. (A) Yeast Hsp90 dimer in complex with co-chaperone p23/Sba1 and ATP analogue (Ali et al., 2006). PDB accession number 2CG9. (B) Amino acids 1–554 of bovine Hsc70 lacking the 10 kDa C-terminal domain (Jiang et al., 2005). PDB accession number 1YUW. (C) The bacterial type I chaperonin (Hsp60 family) GroEL consisting of two stacked homoheptameric rings (Braig et al., 1994) viewed from above one ring. PDB accession number 1GR1. (D) Hexameric structure of an archaeal homologue of prefoldin (Siegert et al., 2000). PDB accession number 1FXK. (E) Dodecameric structure of Hsp16.3 from Mycobacterium Tuberculosis (Kennaway et al., 2005) showing dimer subunits. PDB accession number 2BYU. (F) The J-domain of human Hsp40 family member HDJ-1 (Qian et al., 1996). PDB accession number 1HDJ. (G) Structure of the calnexin luminal domain (Schrag et al., 2001). PDB accession number 1JHN. In all structures α-helices are shown in blue and β-sheets in red. Scale bar represents 10 angstroms.

More than 20 different families of proteins have chaperone activity. The major chaperone families include the Hsp90, Hsp70, Hsp60 (chaperonins), Hsp40 (DnaJ) and small heat shock proteins (sHsps). These different chaperone families provide different structural solutions to the problems of protein folding (Fig. 1). The best-studied and mechanistically understood chaperone machines are Hsp70 and Hsp60 families. These chaperones recognize and interact with unfolded or partially folded polypeptides by binding to exposed hydrophobic regions within proteins preventing them from aggregating and maintaining them in a folding competent state until release. This is particularly important as the nascent polypeptide emerges from the ribosome (Kaiser et al., 2006). Efficient folding of a newly synthesized polypeptide chain is achieved by a transient interaction with the chaperone that prevents aggregation due to unwanted interactions with hydrophobic regions of other proteins or within the extending polypeptide (Dobson, 2004).

The different families of chaperone proteins recognize various intermediates of non-native polypeptides and interact through different modes of binding (Fig. 1). Hsp70 proteins bind to short regions of peptides with a certain position and pattern of hydrophobic residues in a substrate-binding pocket resembling a ‘clamp’ (Jiang et al., 2005). In contrast, Hsp60 (chaperonins) facilitate folding by enclosing non-native polypeptides in the central cavity (or ‘cage’) of a ring structure formed from identical or closely related rotationally symmetrical subunits (Rye et al., 1999). Another example of chaperone activity is the peptidyl–prolyl *cis*–*trans* isomerase (PPIase) activity found in the cyclophilins (e.g. NinaA) that overcomes a rate limiting step in protein folding, the correct orientation of proline residues (Yaffe et al., 1997; Andreotti, 2003).

Molecular chaperones are a group of functionally related but otherwise diverse protein families. These families are conserved among the vast majority of prokaryotic and eukaryotic organisms and members of each family occupy both cytosol and endoplasmic reticulum (ER). The following is a brief description of the properties of these families.

### 1.2 Major chaperone families

#### 1.2.1 Hsp90

Members of the Hsp90 family of chaperones are present in most cellular compartments such as the cytosol, the ER, mitochondria and the chloroplast in plants. Hsp90 functions as a homodimer, and can be divided into three domains, an N-terminal ATPase domain, a ‘middle’ domain and a C-terminal domain. The structure of the N-terminal domain was the first to be solved (Prodromou et al., 1997) and revealed an atypical ATP binding pocket, resolving the controversy over whether Hsp90 was capable of nucleotide binding. The ATP binding pocket is the site of binding for the macrocyclic antitumor agent geldanamycin (Prodromou et al., 1997). Geldanamycin binding to Hsp90 can disrupt productive complexes with protein kinase and steroid hormone receptor clients (Mimnaugh et al., 1996). The action of geldanamycin and related compounds is attributed to their action as competitive inhibitors of ATP binding which is essential for Hsp90 function (Obermann et al., 1998). Recently, the ‘closed’ structure of an Hsp90 dimer in contact with the co-chaperone p23 was solved and revealed that Hsp90 does not enclose its client proteins but provides a bipartite binding surface whose formation and disruption are coupled to the chaperone ATPase cycle (Ali et al., 2006; Fig. 1). Hsp90 fulfils its chaperone functions at the core of a multiprotein complex that incorporates other chaperones and an assortment of co-chaperones. Many Hsp90 co-chaperones contain a tetratricopeptide repeat (TPR) domain but other than this structural feature they are largely different. TPR containing co-chaperones interact with the C-terminus of Hsp90 and facilitate diverse functions (Prodromou et al., 1999). For example, the E3 ubiquitin ligase CHIP functions in targeting Hsp90 client proteins to the proteasome (Jiang et al., 2001).

#### 1.2.2 Hsp70

The Hsp70 family has several members that are both stress-inducible (Hsp70, Hsp70i) and constitutively expressed (Hsc70). There are forms in most cellular compartments including cytoplasm and nucleus (Hsc70), mitochondria (Hsp75, mortallin or mtHsp70), and endoplasmic reticulum (Grp78 or BiP) (Mayer and Bukau, 2005). Hsp70 proteins have an N-terminal ATPase domain and a C-terminal substrate binding (or client binding) domain (Fig. 1). ATP hydrolysis in the N-terminal domain is linked to a conformational change in the client binding domain (Vogel et al., 2006). The client protein binding domain has a base of beta strands and a ‘lid’ that is closed upon ATP hydrolysis to form a ‘clamp’ (Jiang et al., 2005). This clamp binds short extended hydrophobic regions within client proteins and prevents them from aggregation (Jiang et al., 2005). Hsp70 proteins can also bind apparently native proteins, such as the un-coating of clathrin from clathrin cages (Ungewickell et al., 1995). The versatility displayed by the Hsp70 family is achieved through extensive employment of co-chaperones, including Hsp40 proteins, and a range of nucleotide exchange factors (NEFs). TPR containing co-chaperones also bind Hsp70, e.g. Hop (Scheufler et al., 2000).

#### 1.2.3 Hsp60

The Hsp60 family, also known as the chaperonins, form large ring assemblies that assist protein folding (Fig. 1). Two classes of chaperonin have been identified: type I and type II. Type I chaperonins are similar to bacterial GroEL and are composed of two seven-membered rings that are usually homomeric. These are found in mitochondria (Hsp60), chloroplasts (Rubisco binding protein), and the eubacterial cytosol (GroEL). Type II chaperonins are still formed of two rings but the rings are more diverse and are usually composed of several different subunits. In archaebacteria the type II chaperonin is known as the thermosome and the chaperonin of the eukaryotic cytosol is known as CCT or TRiC (Horwich et al., 2007). These chaperonins have co-chaperones (or co-chaperonins), such as GroES for GroEL and phosducin-like proteins for CCT. GroEL and GroES function together to form a ‘two-stroke engine’ for protein folding that is regulated by ATP binding, hydrolysis and release in the different rings (Rye et al., 1999). Client proteins are bound within a central cavity, GroES binds and upon ATP hydrolysis the clients are released into the now encapsulated cavity to fold productively before finally being released as a native protein or rebound by the chaperone system.

#### 1.2.4 Hsp40 (DnaJ)

The Hsp40 or DnaJ family of chaperones are defined by the presence of a highly conserved 70 amino acid J-domain, which is found at the N-terminus of *Escherichia coli* DnaJ (Pellecchia et al., 1996). The J-domain has two anti-parallel alpha helices joined by a loop that contains the HPD motif and two other alpha helices that stabilize the structure (Fig. 1). Outside of this J-domain, Hsp40 proteins can be extremely diverse and have been classified on the basis of their domain conservation with DnaJ as type I, full domain conservation; II, conservation of N-terminal J-domain and neighboring G/F domain; or III, J-domain alone (Cheetham and Caplan, 1998). Consequently, the Hsp40 family has a wide range of molecular weights from less than 20 to over 500 kDa. This is probably the most diverse and numerous of the chaperone families with over 50 different human proteins that contain a J-domain or J-like domain, which has imperfect conservation of an HPD motif within the J-domain. The J-domain mediates the interaction of Hsp40 proteins and their partner Hsp70s (Hennessy et al., 2005).

#### 1.2.5 Small Hsps (sHsps)

The molecular mass of these chaperones ranges from 15 to 42 kDa monomers that come together to form dynamic oligomeric structures, based around dimer subunit assembly (Haslbeck, 2002). The major lens proteins the α-crystallins are members of this family of chaperones. The sHsps share a conserved ‘α-crystallin domain’ of approximately 90 residues in the C-terminal part of the protein (Haslbeck, 2002). These chaperones are not thought to be regulated by nucleotide binding and release but their function and oligomerization are regulated by phosphorylation and temperature. For example, the dimer of Hsp26 specifically recognizes and binds unfolded polypeptide chains (Haslbeck et al., 1999). Following phosphorylation, the oligomeric structure of sHsps undergoes changes. Phosphorylation of Hsp27 leads to its dissociation and reduced chaperone activity (Rogalla et al., 1999). In contrast Hsp25 showed chaperone activity as a hexadecamer (Ehrnsperger et al., 1999). sHsps do not appear to have dedicated co-chaperones but instead seem to network with other chaperone families.

#### 1.2.6 Calnexin and lectin chaperones

The ER uses the glycosylation of proteins in conjunction with lectin chaperones to monitor protein folding and enhance quality control. Calnexin is a type I transmembrane protein that interacts with monoglucosylated intermediates bearing the sugar appendage Glc_1_Man_7–9_GlcNAc_2_. Calnexin has a soluble homologue, calreticulin, that is resident in the ER lumen. Nascent glycoproteins associate with calnexin and calreticulin through cycles of binding and release (Molinari et al., 2004). This cycle is essential for quality control as its exposes unfolded glycoproteins to folding factors and accessory enzymes, which direct correct protein folding (Ellgaard and Helenius, 2003). ERp57 is one of these factors thought to be recruited to folding glycoproteins through its interaction with calnexin or calreticulin (Lindquist et al., 2001) although ERp57 may also function as an unassisted chaperone (Peaper et al., 2005).

### 1.3 Regulation of molecular chaperone function

**Fig. 2:**
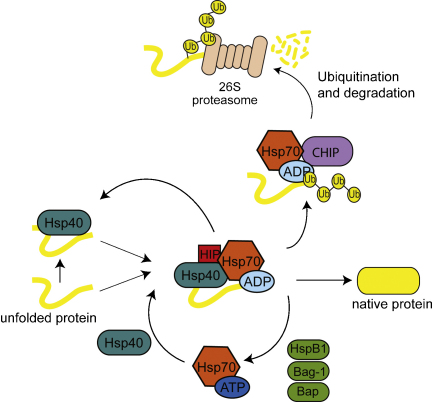
Co-chaperone regulation of chaperone function. Schematic diagram of the Hsp70 nucleotide based reaction cycle. In this model, the client protein is presented to Hsp70 by the Hsp40 co-chaperone, or Hsp70 is activated by Hsp40 to bind a client nearby. The bound client can be stabilized on Hsp70 by the Hsp70 interacting protein (Hip) or release stimulated by a nucleotide exchange factor, such as Bag1 or HspBP1. The released client protein may fold to the native state or enter another cycle of chaperone binding and release. If the client spends too long in the chaperone system, or if it is targeted by specialized co-chaperones, it will be sorted for degradation by C-terminus of Hsp70 interacting protein (CHIP) and subsequently degraded by the proteasome (Hohfeld and Jentsch, 1997; Hennessy et al., 2005).

For many chaperones, cycles of client protein binding and release are coupled to conformational changes in the chaperone protein that are dependent on the hydrolysis and exchange of ATP. This process is regulated by protein cofactors known as co-chaperones. Co-chaperones function synergistically with the major chaperones in protein folding and often have independent chaperone activity, but their major role may be to provide these folding machines with specificity in client protein binding. For example, the Hsp70 protein machinery achieves its multiple cellular functions because of various co-chaperone proteins, such as the Hsp40 (DnaJ) family that stimulate Hsp70 ATP hydrolysis through their conserved J-domain and other co-chaperones that regulate nucleotide exchange (Fig. 2). In this model, the client protein is presented to Hsp70 by the Hsp40 co-chaperone, or Hsp70 is activated by an Hsp40 to bind a client nearby (Cheetham and Caplan, 1998). The bound client can be stabilized on Hsp70 by the Hsp70 interacting protein (Hip) or its release can be stimulated by an exchange factor, such as Bag1 or HspBP1 (Hohfeld and Jentsch, 1997; Kabani et al., 2002). The released client protein may fold to the native state or enter another cycle of chaperone binding and release. If the client spends too long in the chaperone system or if it is targeted by specialized co-chaperones it will be sorted for degradation by the proteasome (Westhoff et al., 2005).

In addition to the J-domain there are some other common co-chaperones motifs. For example, Hip (Hohfeld and Jentsch, 1997), Hop (Scheufler et al., 2000), CHIP (Connell et al., 2001) and the immunophilins, including AIPL1 (Sohocki et al., 2000), use a degenerate 34 amino acid repeat motif, the TPR to promote chaperone/co-chaperone interactions and modify chaperone function.

In addition, there is functional co-operation between the individual chaperone machines. For example, the Hsp70/Hsp90 organizing protein (Hop) functions to bring together these two chaperone machines to form a larger chaperone heterocomplex (Scheufler et al., 2000). This chaperone heterocomplex of Hsp70, Hsp90 and several co-chaperones functions in the assembly of the steroid receptor and transcription factor complexes (Morishima et al., 2000). Similarly, the Hsp70 chaperone machine may pass nascent chains onto Hsp60 to complete folding (Frydman et al., 1994) and sHsps may sequester denatured proteins preventing them from aggregation until another chaperone system can facilitate their folding or degradation (Lee et al., 1997; Lee and Vierling, 2000). Through these complementary but distinct roles in protein folding, molecular chaperones can facilitate changes in protein conformation from initial folding through function and ultimately to degradation. These protein folding machines can also be finely tuned to fulfil specific roles within cells. The highly specialized photoreceptor cell, like all other cells, uses the chaperone machinery to perform fundamental cellular functions and it also exploits the capacity for specialization to meet its own specific biology.

## 2 Molecular chaperones in retinal degeneration

The neural sensory retina is a highly specialized part of the CNS. Inherited retinal dystrophies are characterized by a great degree of phenotypic and genetic heterogeneity with over 120 different disease genes implicated (RetNet, http://www.sph.uth.tmc.edu/RetNet/). Mutations in many of these essential retinal genes can result in protein misfolding with subsequent loss of normal protein function. The misfolded protein can also acquire new, toxic gains of function thought to be associated with aggregation of the misfolded protein. Loss of protein function is usually associated with disease gene mutations resulting in recessive disease or haplo-insufficient dominant disease, whereas other dominant disease mechanisms will be associated with gain of function or dominant negative effects of genetic mutations. In this review, we will focus on how advances in the genetics and pathogenesis of inherited retinal disease have revealed specialized roles for molecular chaperones in the light sensitive photoreceptor cells. In particular, we will focus on the role of chaperones in response to protein misfolding disease and mutations in putative chaperones that cause retinal degeneration.

### 2.1 Mutations in rhodopsin cause retinitis pigmentosa

Retinitis pigmentosa (RP) is a genetically heterogeneous group of diseases that converge in their symptoms and in the manner in which the retina is affected. Patients present with night blindness and loss of peripheral vision, as the rod photoreceptor cells dysfunction and die followed by cone photoreceptor cell death. The disease then progresses towards the centre of the retina, leading to characteristic tunnel vision and eventual blindness. RP has multiple modes of inheritance: 15–20% of all cases are autosomal dominant (ADRP), 20–25% are autosomal recessive (arRP), 10–15% are X-linked (XLRP), and the remaining 40–55% cannot be classified genetically (Wang et al., 2005; Daiger et al., 2007). Examples of retinal disease genes that have been associated with protein misfolding are summarized in Table 1. The best studied of these is the rod cell light sensitive photopigment, rhodopsin, which is mutated in approximately 30% of cases of ADRP, (Wang et al., 2005) making it one of the most common causes of RP. Rhodopsin is comprised of the rod opsin protein and the chromophore 11-*cis*-retinal. Over 140 mutations have been identified in rod opsin (RetNet, http://www.sph.uth.tmc.edu/RetNet/). These mutations have been classified according to their cellular or biochemical properties (reviewed by Stojanovic and Hwa, 2002; Mendes et al., 2005). Of particular interest to this review are the class II mutants that lead to rod opsin protein misfolding. Class II is the most common class of rod opsin mutations and includes P23H, which is also the most common cause of ADRP in North America. Dryja et al. (1990) first identified the P23H mutation. The proline at position 23 is highly conserved among vertebrate and invertebrate opsins and in other G-protein coupled receptors, for example beta-2-adrenergic receptor (Applebury and Hargrave, 1986). Similarly, many other residues affected by class II mutations are conserved and reside in the intradiscal and transmembrane domains that are critical to the correct folding of the protein (Mendes et al., 2005).

### 2.2 Class II rod opsin protein misfolding and aggregation

**Fig. 3:**
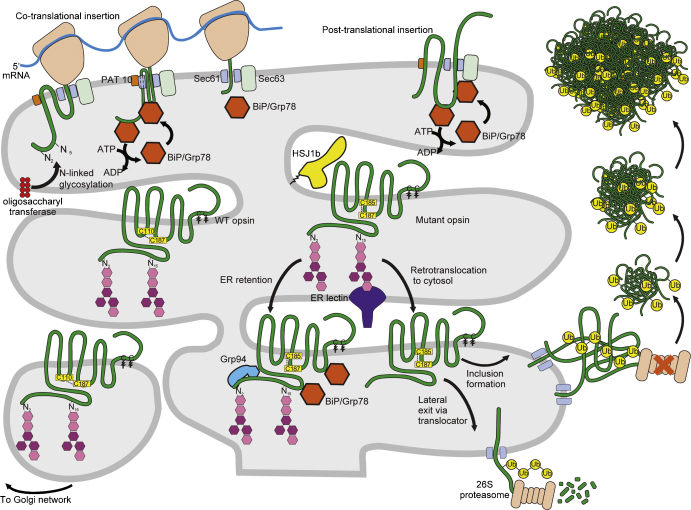
The role of chaperones in rod opsin biogenesis, quality control and degradation. Schematic representation of rod opsin folding and misfolding. Rod opsin does not encode a cleaved amino-terminal signal sequence, but is still thought to enter the secretory pathway by co-translational or post-translational insertion into the ER via the ER translocon. The ER translocon is thought to align with the large ribosomal subunit to facilitate entry and prevent flow of ions from the ER. The ER Hsp70, BiP, located in the luminal side, assists with protein insertion by a ‘ratchet mechanism’ coupled to ATP hydrolysis. The Hsp40 protein Sec63 interacts with BiP through its J-domain and modulates BiP function (Misselwitz et al., 1998). Properly folded rod opsin, with the correct disulphide linkage (Hwa et al., 1999) will translocate to the Golgi for further processing. In contrast, mutant opsin is more likely to misfold and form the wrong disulphide bond (Hwa et al., 1999) and will be retained in the ER by resident chaperones such as Grp94 or BiP (Anukanth and Khorana, 1994). The misfolded rod opsin may also interact with HSJ1b in the cytoplasm and a lectin chaperone in the ER lumen. Mutant opsin is degraded by the proteasome in the cytoplasm following ERAD. If the ERAD machinery is overwhelmed, mutant opsin aggregates upon dislocation in the cytosol (Illing et al., 2002; Saliba et al., 2002). These aggregates in turn set up a positive feedback loop by inhibiting the proteasome and enhancing aggregation (Illing et al., 2002). The aggregated opsin can coalesce into large aggregates, eventually forming visible inclusions. These inclusions and aggregates sequester cellular proteins, including molecular chaperones such as Hsp70 and HSJ1.

A wealth of data from animal models and cell biological studies has shown that class II rod opsin mutations shift the folding equilibrium away from the native state and towards folding intermediates that have a propensity to misfold and aggregate. In animal models, wild-type (WT) rhodopsin is almost entirely restricted to the outer segment, a specialized rod photo-sensing organelle. In contrast, class II rod opsin mutants show abnormal accumulation in the photoreceptor outer nuclear layer, outer plexiform layer and are also detected in inner and outer segments (Roof et al., 1994; Frederick et al., 2001). In cultured cells, WT opsin translocated to the plasma membrane, whereas class II mutants were retained in the ER, and could not be reconstituted with 11-*cis*-retinal suggesting protein misfolding (Sung et al., 1991, 1993; Kaushal and Khorana, 1994). The misfolded class II rod opsin is retrotranslocated from the ER to the cytosol and degraded by the ubiquitin–proteasome system or can aggregate into cytosolic aggregates (Illing et al., 2002; Saliba et al., 2002). These rod opsin aggregates can coalesce into large inclusions (Fig. 3) with the properties of an aggresome similar to those described for several polytopic and monotopic integral membrane proteins (Johnston et al., 1998).

In addition to gain of function mechanisms, misfolded opsin can act as a dominant negative to affect the processing and fate of the wild-type protein. Studies on *Drosophila* Rh1 (Kurada et al., 1998) and mammalian rod opsin in cells (Saliba et al., 2002; Rajan and Kopito, 2005) suggested that misfolded rod opsin had a dominant effect on the WT protein. When both WT and mutant rod opsin were present, as in patients heterozygous for ADRP, P23H was found to affect the processing of WT opsin, causing the wild-type protein to aggregate and form inclusions (Saliba et al., 2002). Furthermore, the mutant and WT opsins appeared to form high molecular weight, detergent insoluble complexes, in which the two proteins were in close (<70 Å) proximity (Rajan and Kopito, 2005).

Rod opsin misfolding and aggregation has many similar features to the protein misfolding and aggregation observed in other neurodegenerative diseases such as familial amyotrophic lateral sclerosis (ALS) (Matsumoto et al., 2006), polyglutamine disease (Gidalevitz et al., 2006) and prion disease (Collinge and Clarke, 2007). For example, similar to the inclusions in many neurodegenerations, P23H-opsin inclusions recruit ubiquitin and Hsp70 chaperones (Saliba et al., 2002). Nevertheless, the basis of toxicity of class II rhodopsin mutations remains to be elucidated but it is likely that similar gain of function mechanisms apply as in other neurodegenerations (see Mendes et al., 2005 for more detail). Therefore, it is important that we consider the role of chaperones in opsin biogenesis and their role in opsin quality control and degradation.

### 2.3 Rod opsin interacts with multiple ER chaperones during biogenesis

The biogenesis and quality control of multi-spanning membrane proteins occurs at the ER. Certain steps in this pathway and the potential involvement of molecular chaperones are shown schematically in Fig. 3. The rod opsin signal sequence binds to the signal recognition particle (Audigier et al., 1987) directing the ribosome and the growing polypeptide to the ER membrane. This signal sequence is not cleaved (Friedlander and Blobel, 1985) and opsin inserts in the ER co-translationally (Kanner et al., 2002). The rod opsin transmembrane domains are thought to undergo active and passive displacement from the Sec61 translocon into the lipid bilayer (Ismail et al., 2006). PAT-10, a transmembrane specific chaperone, associates with the TM1 of rod opsin until the entire polypeptide chain has been translocated, either facilitating insertion or modulating the assembly of individual transmembrane domains (Meacock et al., 2002).

Upon insertion into the ER membrane the N-terminal intradiscal domain of rod opsin is N-glycosylated at Asn_2_ and Asn_15_ by the oligosaccharyl transferase enzyme (Plantner et al., 1980; Kean, 1999). Glycans are known to be required for the association with lectin chaperones that can either assist in polypeptide folding or ER-associated degradation (ERAD) of a terminally misfolded protein. Glycan chains are required for efficient ERAD of misfolded rod opsin (Saliba et al., 2002). These data imply a role for lectin chaperones such as calnexin, calreticulin or EDEM in opsin quality control, but the specific lectin chaperones that regulate opsin ERAD remain to be identified. Other chaperones may also regulate opsin quality control. For example, Anukanth and Khorana (1994) showed that mutant opsins were found in a complex with the Hsp70 and Hsp90 chaperones of the ER, namely Grp78 (BiP), and Grp94.

An increase in BiP mRNA levels was induced in cells by P23H rod opsin expression compared to WT rod opsin or GFP expression, suggesting that the protein misfolding associated with class II rod opsin can induce the unfolded protein response (UPR) (Lin et al., 2007). However, in transgenic P23H rats, BiP mRNA levels decreased as the retina degenerated, whereas CHOP mRNA was induced (Lin et al., 2007). These data suggest that photoreceptors may be unable to adapt to the production of P23H rod opsin by upregulating their ER resident chaperones via the UPR. Sustained signaling through the PERK branch of the UPR is associated with the induction of the proapoptotic factor CHOP which may lead to cell death. Therefore, a failure of the chaperone machinery to cope with misfolded opsin may directly lead to photoreceptor cell death.

Specific lectins for the maturation and processing of mammalian rod opsin have not yet been identified. Calnexin is required, however, for the maturation of the *Drosophila* rod opsin protein, Rh1, a homologue of mammalian rod opsin (Rosenbaum et al., 2006). Flies with premature stop codons in their *cnx* gene displayed reduced levels of *cnx* transcript with no detectable calnexin protein. These mutations severely reduced Rh1 protein levels, suggesting that Rh1 degradation was taking place. Furthermore, *cnx* mutant flies displayed age-related retinal degeneration as shown by reduced translocation of Rh1 to rhabdomeres 1–6 and reduced rhabdomere size. Calnexin also formed a stable complex with Rh1, consistent with its role as molecular chaperone (Rosenbaum et al., 2006). There may be, however, significant differences between Rh1 and mammalian rod opsin in this respect. An intact carbohydrate unit does not appear to be essential for the chromophoric properties of rhodopsin or for its regeneration (Renthal et al., 1973). Furthermore, Kaushal et al. (1994) showed that treatment of WT opsin with the glycosylation inhibitor, tunicamycin, resulted in non-glycosylated opsin, which translocated to the cell surface and formed the characteristic rhodopsin chromophore with 11-*cis*-retinal. On the other hand, treatment of P23H opsin expressing cells with tunicamycin led to P23H accumulation in the ER, suggesting that glycans are required for mutant rod opsin ERAD (Saliba et al., 2002). Other proteins that undergo ERAD have been shown to dislocate to the cytosol where they are deglycosylated by cytosolic N-glycanases, suggesting that the carbohydrates are recognized by lectins that mediate and process the misfolded polypeptides for degradation by the proteasome (Wiertz et al., 1996a, b; Petäjä-Repo et al., 2001; Gong et al., 2005). The actual targeting into ERAD of misfolded opsin could be mediated by chaperones that may not interact with normal folding intermediates. This is the case for the cystic fibrosis transmembrane conductance regulator (CFTR) where Hsp90 is required for the folding of its large cytoplasmic domains but Hsp70 and Hsp40 are required for targeting it for degradation (Loo et al., 1998; Youker et al., 2004; Zhang et al., 2006).

Other evidence suggests that Rh1 and mammalian opsin may have different chaperone requirements. NinaA is an ER resident integral membrane peptidyl–prolyl isomerase (PPI) chaperone that is required for the maturation of Rh1 rhodopsin. NinaA mutant flies have very little Rh1 opsin in their rhabdomeres, with most of Rh1 immunoreactivity associated with ER membranes (Colley et al., 1991; Stamnes et al., 1991; Baker and Zuker, 1994). These findings suggest that NinaA activity is required for the transport of Rh1 from the ER. Furthermore, examination of NinaA mutants revealed reduced levels of rhodopsin and large accumulations of rough ER membranes (Colley et al., 1991). In contrast, heterologous expression of bovine rod opsin in *Drosophila* resulted in correctly folded and processed rod opsin even in the absence of NinaA (Ahmad et al., 2006). The reasons for this difference in chaperone requirement remains to be elucidated but may help in clarifying the role of chaperones in mammalian opsin biogenesis.

### 2.4 Non-ER chaperone opsin interactions

The retina is rich in chaperones and several could be involved in rod opsin biogenesis or the response to rod opsin misfolding. For example, almost 20 different crystallin genes have been identified in the retina (Xi et al., 2003). The crystallins can be divided into two major families, α and βγ with α-crystallins being members of the sHsp family of molecular chaperones. In the retina, α-crystallin co-purified with post-Golgi membranes from frog photoreceptors and, therefore, could be involved in the transportation of newly synthesized rhodopsin, suggesting that these chaperones might participate in the renewal of the photoreceptor outer segment (Deretic et al., 1994).

In addition, specialized Hsp40 (DnaJ) proteins are expressed at relatively high levels in the retina compared to other tissues. HSJ1a and HSJ1b are neuronal Hsp40 proteins that are present in all layers of the neural retina and have distinct staining patterns, with HSJ1b found to be enriched at the site of rhodopsin production (Chapple and Cheetham, 2003). Heterologous expression in cell culture showed that the HSJ1b isoform increased the incidence of WT and P23H rod opsin inclusions, and increased the retention of the WT protein in the ER. This was not observed with HSJ1a or the C321S HSJ1b prenylation null mutant (Chapple and Cheetham, 2003). These Hsp40 proteins act as neuronal shuttling factors for the sorting of chaperone clients to the proteasome (Westhoff et al., 2005). HSJ1 proteins colocalized with rod opsin in inclusions, suggesting that they may be part of the opsin degradation pathway. The full significance of the interaction of HSJ1 proteins with rod opsin remains to be determined; however, it is clear that the retina has specialized chaperones that are essential for normal vision.

### 2.5 Chaperone inducers and retinal protein misfolding disease

It is now widely accepted that chaperones can act as protectors of the proteome and may be manipulated to combat protein misfolding diseases. The over-expression of molecular chaperones can extend life span in the nematode and fruit fly (Tatar et al., 1997; Hsu et al., 2003). Molecular chaperones have been shown to suppress aggregate formation of mutant proteins that cause neurodegenerative diseases, such as spinocerebellar ataxia 1 (SCA1) (Cummings et al., 1998), spinal and bulbar muscular atrophy (SBMA) (Kobayashi et al., 2000), familial amyotrophic lateral sclerosis (FALS) (Takeuchi et al., 2002) and Huntington disease (Jana et al., 2000; Westhoff et al., 2005). Therefore, it may be possible to alleviate protein misfolding diseases by enhancing the expression of molecular chaperones using drugs. A chaperone inducer is a drug that can activate heat shock transcription factors (HSFs) and induce chaperone expression. Several drugs that can induce chaperones are now in clinical trials. For example, drugs based around the Hsp90 inhibitor geldanamycin, such as 17-allylamino-17-demethoxygeldanamycin (17-AAG), induce HSF activity. In a cell culture model of rhodopsin RP based on P23H rod opsin, treatment with 17-AAG led to a dose-dependent reduction of inclusion incidence. This correlated with an increase in cell viability and a reduction in caspase activation (H.F. Mendes and M.E. Cheetham, under review). Therefore, manipulation of chaperones either by drugs or by gene manipulation may be useful to treat photoreceptor protein misfolding diseases.

## 3 Putative chaperones as retinal disease genes

In the last 10 years the expanding list (http://www.sph.uth.tmc.edu/Retnet/home.htm) of retinal disease genes has identified several putative chaperone proteins as being essential either in the development or maintenance of a functional retina. These chaperones and putative chaperones are shown in Table 2. In contrast to ADRP, all of these diseases are caused by loss of protein function, highlighting that a lack of chaperone function can be critical to many aspects of photoreceptor cell biology.

### 3.1 RP2

X-linked RP (XLRP) is generally associated with the most severe forms of RP. Mutations in the *RP2* gene account for approximately 15% of all XLRP cases (Schwahn et al., 1998; Hardcastle et al., 1999; Miano et al., 2001). The RP2 protein is widely expressed in human tissues and does not appear to be enriched in retina (Chapple et al., 2000). As patients with *RP2* mutations appear to have only retinal pathology without any other organ involvement, it is not clear why the loss of RP2 leads specifically to RP. RP2 is a putative chaperone based on its homology to a previously identified chaperone in the tubulin folding pathway. RP2 is a 350 amino acid protein, which contains a homology domain of 151 amino acids (30% identity) to the tubulin cofactor C (TBCC). Most of the reported RP2 missense mutations correspond to this region and lie within the residues conserved with TBCC.

TBCC, together with other specific cofactor chaperones (tubulin cofactors A–E, TBCA, TBCB, TBCD and TBCE), acts downstream of the cytosolic chaperonin CCT in the tubulin folding pathway (Tian et al., 1996). Tubulin heterodimers cannot form without the assistance of molecular chaperones (Lewis et al., 1992; Yaffe et al., 1992). Once quasi-native α and β tubulin have been released from CCT, these cofactors facilitate the assembly of the α/β tubulin heterodimer and stimulate the GTPase activity of the β tubulin within the heterodimer, leading to the formation of native tubulin heterodimers (Tian et al., 1999). This process is regulated by ADP ribosylation factor-like 2 (Arl2) (Bhamidipati et al., 2000; Shern et al., 2003). Correct expression of the tubulin cofactors TBCC, TBCD and TBCE is vital, as mutations in these genes lead to a lack of microtubules and failure of cell division in Aradopsis (Steinborn et al., 2002, Kirik et al., 2002). In addition, TBCD also functions as a centrosomal protein. Over expression of TBCD in HeLa cells lead to the loss of gamma tubulin at the centrosomes and cells appeared compromised in their ability to nucleate microtubules. Whereas loss of TBCD caused failure of cytokinesis and an increase in cells with mitotic spindle defects (Cunningham and Kahn, 2008).

The similarity between RP2 and TBCC suggested a potential overlap in function. This hypothesis was supported by *in vitro* studies that demonstrated RP2, in conjunction with TBCD, could partly substitute for TBCC function as a tubulin-GTPase activating protein (GAP) (Bartolini et al., 2002). Furthermore, a pathogenic mutation in the conserved arginine residue, R118H in RP2, abolished the tubulin GAP activity in both RP2 and cofactor C, suggesting that this residue could be an ‘arginine finger’, which triggers the tubulin GAP activity. However, RP2 was not able to replace TBCC in the tubulin heterodimerization reaction (Bartolini et al., 2002). Similarly, RP2 cannot be functionally substituted by TBCC, as TBCC does not compensate for RP2 in rods of XLRP patients (Grayson et al., 2002).

There are several clear differences between RP2 and TBCC. For example, RP2 is subject to posttranscriptional modification by myristoylation and palmitoylation at its N-terminus, which target RP2 to the plasma membrane (Chapple et al., 2000, 2002, 2003; Grayson et al., 2002). RP2 acylation is disrupted by a patient mutation deletion of serine 6 (ΔS6), which prevents the correct sub-cellular targeting of RP2. Therefore, aberrant post-translational modification and localization of RP2 might cause RP in these patients (Chapple et al., 2000). Furthermore, the C-termini of RP2 and TBCC are not similar. The C-terminus of RP2 shows structural similarity with nucleoside diphosphate kinase 1 (NDK1) (Evans et al., 2006; Kuhnel et al., 2006). NDKs catalyze the transfer of phosphates, mainly from ATP to cognate NDPs generating nucleoside triphosphates (NTPs), and can also have autophosphorylation activity from ATP and GTP (reviewed by Hasunama et al., 2003). Previously, NDK was shown to interact with Hsp70 (Leung and Hightower, 1997), while Hsp70, similar to NDK, exhibits an intrinsic ADP–ATP phosphorylation activity (Hiromura et al., 1998). Therefore, functionality of the RP2 C-terminal NDK domain could provide further evidence for molecular chaperone activity. RP2 does not have nucleoside diphosphate kinase activity, but it does share exonuclease activity with NDK 1 and 2 (Yoon et al., 2006). Furthermore, RP2 translocated to the nucleus upon induced DNA damage, potentially linking RP2 with the cellular stress response machinery (Yoon et al., 2006).

The only identified interaction partner of RP2 is the ADP ribosylation factor (Arf)-like protein Arl3 (Bartolini et al., 2002). The affinity of RP2 to GDP bound Arl3 is 400-fold weaker than to GTP-bound Arl3, indicating that RP2 is a *bona fide* effector of Arl3 (Kuhnel et al., 2006). Myristoylation of RP2 also decreases this affinity, leading to the proposal that Arl3 might interact with unmodified RP2 and facilitate RP2 targeting for acylation (Chapple et al., 2000; Bartolini et al., 2002; Kuhnel et al., 2006). Arl3 is a ubiquitous microtubule associated protein (MAP) and localized to the connecting cilium in photoreceptors (Grayson et al., 2002). The connecting cilium functions as a specialized sensory axoneme that extends from the cell body of the photoreceptor to form the outer segment. The link between retinal degeneration and dysfunction of this cilium has become increasingly evident, as many RP causing proteins, such as the retinitis pigmentosa GTPase regulator (RPGR), have been localized to cilia and shown to be essential for cilia function. RPGR is another major cause of XLRP (Meindl et al., 1996, reviewed by Adams et al., 2007) and RPGR is anchored to the connecting cilium by another retinal disease gene product the RPGR-interacting protein (RPGRIP), a structural component of the ciliary axoneme (Hong et al., 2001). Interestingly, Arl3 interacts with the phosphodiesterase delta (PDEδ), which is also an interacting partner of RPGR (Linari et al., 1999a, b). The significance of these protein interactions remains to be clarified.

Arl3 may have other cytoskeletal functions, especially as the protein is present at microtubule-rich structures, such as centrosomes, mitotic spindle and midbodies, in a wide variety of cell lines. In addition, Arl3 knock-down in HeLa cells caused increased acetylation of alpha-tubulin, leading to failure of cytokinesis and an increased number of binucleated cells (Zhou et al., 2006).

As yet, Arl3 has not been shown to cause retinal dystrophy in humans, but studies in mice deficient in Arl3, revealed that this protein is essential for photoreceptor and kidney development (Schrick et al., 2006). Arl3 knock-out mice showed abnormal epithelial cell proliferation and cyst formation in kidney, liver and pancreas, in the retina there was photoreceptor degeneration (Schrick et al., 2006). Overall, this phenotype is consistent with a failure of function or signal transduction in primary cilia. The formation of cilia is coordinated with progression through the cell cycle via specific signaling pathways and there is growing evidence that defects in these pathways cause proliferative diseases, such as cystic disorders in kidney, liver and pancreas (reviewed by Quarmby and Parker, 2005). The link between Arl3 and cilia function, however, strengthen the notion that RP2 may be required for specialized elements of the cytoskeleton in photoreceptors.

Further evidence for a relationship of RP2 and cilia function was obtained when a homologue of TBCC and RP2 was identified in trypanosomes (TbRP2). Ablation of TbRP2 caused shortened flagella and defective axonemal microtubule formation, but had no effect on other microtubule structures or functions including spindle formation. TbRP2 localized to the mature basal body of the flagellum, suggesting flagellum specific functions of the protein. One of these functions appears to be the recruitment and possibly monitoring the quality of carboxyl-tyrosinated alpha tubulin prior to cilia assembly (Stephan et al., 2007). These findings suggest that RP2 may cause XLRP through problems in assembly and chaperoning of components of the primary cilia. However, there are striking structural differences between the RP2 protein in humans and trypanosomes. For example, TbRP2 lacks the C-terminal NDK domain, as well as the N-terminal dual acylation motif, both of which are likely to contribute to RP2 function as patient mutations compromise these motifs. These differences might be crucial to explain why the RP2 phenotype is restricted to the retina, despite the ubiquitous expression of the protein and importance of cilia in other organs. The importance of specialized chaperones in cilia function, however, is now becoming clear through the identification of causative genes in cilia diseases or ‘ciliopathies’.

### 3.2 Bardet–Biedl Syndrome (BBS) proteins

Bardet–Biedl Syndrome is a pleiotropic, genetically heterogeneous disorder caused by defects in primary cilia and/or basal body proteins in several organs. The BBS genes identified to date show limited structural and functional similarities despite the fact that mutations in BBS genes give rise to the same undistinguishable phenotype. Generally patients suffer from retinal dystrophy, renal dysplasia, postaxial polydactyly, obesity and hypogonadism with infertility in males [OMIM 209900].

Since the first BBS gene (BBS6) was identified in 2000 (Slavotinek et al., 2000; Katsanis et al., 2000), 12 genes (BBS1-12) have been identified. BBS1 and BBS10 are the most common BBS loci, accounting for 23–56 and 20% of mutations, respectively (reviewed by Blacque and Leroux, 2006). To date no correlation between genotype and phenotype has been established.

BBS1, BBS2 and BBS7 proteins have predicted beta-propeller domains, which are common motifs implicated in a variety of functions, such as enzyme catalysis, scaffolding and signaling, ligand binding and transport, as well as mediation of protein–protein interactions (Jawad and Paoli, 2002). Interestingly, many beta propeller domain proteins (such as Gβ) are clients for chaperonin based protein folding, presumably because of particular structural requirements of this type of fold (Kubota et al., 2006). Several good animal models of BBS have been produced. For example, the BBS1 (M390R) knock-in mouse model presented with retinal degeneration, decreased olfaction, obesity and lack of sperm flagella, resembling phenotypes of other BBS mouse models. The BBS1 mutant knock-in mice showed severe photoreceptor degeneration with disrupted morphology and orientation of outer segment membranes (Davis et al., 2007). In addition, many BBS patients suffer from hypoosmia or anosmia, indicating lack or dysfunction of olfactory sensory cilia. This hypothesis has been confirmed using mice which lack BBS1 or BBS4. These mice showed a reduction in the olfactory ciliated border, as well as defects in dendritic microtubule organization and trapping of olfactory cilia proteins in dendrites and cell bodies (Kulaga et al., 2004, reviewed by Fliegauf et al., 2007).

Interestingly, the BBS3 gene encodes the Arl6 protein, which like Arl2 and Arl3 is a member of the Arf subfamily of Ras related proteins (Fan et al., 2004). Unfortunately, not much is known about the function of Arl6, but a worm orthologue of the protein is expressed in sensory neurons and is proposed to traffic along cilia by intraflagellar transport (IFT), supporting a role for Arl6 in cilia function (reviewed by Kahn et al., 2005).

In a recent study, seven BBS proteins (BBS1, BBS2, BBS4, BBS5, BBS7, BBS8 and BBS9) were shown to form a biochemically stable protein complex, the ‘BBSome’ (Nachury et al., 2007). This complex transiently associated with pericentriolar material 1 (PCM-1) and alpha tubulin, as well as localizing to the membrane of the primary cilium. Knock-down of BBS1 and BBS5 affected ciliogenesis by interfering with membrane trafficking to the cilium. In addition, Rabin8 a guanosyl exchange factor for Rab8, which facilitates vesicular trafficking between different compartments, was associated with the BBSome. Therefore, BBS proteins may function in membrane trafficking in the primary cilium (Nachury et al., 2007). These data led to the proposal that the genetic complexity of BBS can be reduced to two highly conserved entities, the core BBSome and the small GTPase Arl6/BBS3, while all other BBS genes are limited to chordates and are possibly regulators of the BBSome or Arl6/BBS3 (Nachury et al., 2007). Curiously, the BBS genes excluded from the BBSome, BBS6, BBS10 and BBS12 are all limited to the vertebrate lineage (or possibly chordates for BBS6) and define a divergent branch of chaperonin-like proteins (Kim et al., 2005; Stoetzel et al., 2006, 2007).

Sequence analysis of BBS12 showed that BBS12, together with BBS6 and BBS10 are similar to type II chaperonins. All three proteins have a typical chaperonin-like organization, encompassing the three major functional domains: equatorial, intermediate and apical. However, compared to prototypic type II chaperonins BBS6, BBS10 and BBS12 contain specific insertions in the equatorial and the apical domains. Here BBS12 shares with BBS6 the same insertions in the core chaperonin fold of the equatorial domain and with BBS10 in the apical domain. Given the structural constraints introduced by the additional domains it was predicted that it is unlikely that BBS6, BBS10 and BBS12 are involved in the assembly of a prototypic chaperone complex similar to CCT (Stoetzel et al., 2006, 2007). However, the presence of additional domains within the proteins might confer a specific binding or protein folding capacity to these BBS proteins, which is lacking in other type II chaperonins.

This might be especially important as BBS6 and BBS12 lack several residues critical for ATP binding and hydrolysis, which are important in other chaperonins for protein folding (Kim et al., 2005; Stoetzel et al., 2007). In contrast to BBS6 and BBS12, the functional motif for ATP hydrolysis is conserved in BBS10, suggesting that BBS10 might function as an active enzyme (Stoetzel et al., 2006). These findings highlight an important role of atypical type II chaperonins in the pathogenesis of the disease, since these three BBS genes account for approximately 30% of BBS mutations (Stoetzel et al., 2007).

Suppression of either BBS6, BBS10 or BBS12 in zebrafish yielded gastrulation-movement defects characteristic of other BBS morphants, whereas simultaneous suppression of all three chaperonin-like BBS proteins resulted in severely affected embryos, possibly hinting at a partial functional redundancy within this protein family (Stoetzel et al., 2007).

BBS6 and BBS10 (and possibly BBS12) are centrosomal proteins (Stoetzel et al., 2007) and BBS6 expression is required for cytokinesis (Kim et al., 2005). In addition, knock-down of BBS6 has been implicated in planar cell polarity (PCP) defects (Ross et al., 2005). The fact that BBS6, BBS10 and BBS12 arose only in the vertebrate lineage, whilst architecture and function of the basal body and cilium have been conserved rigorously during evolution might indicate the acquisition of novel cilia functions and/or requirements coincident with the emergence of these particular type II chaperonins. The three BBS proteins might have co-evolved with vertebrate specific proteins, which are involved in basal body and/or cilia assembly and function. Structural analysis of BBS6 revealed that the protein might function as a subunit of a novel hetero-oligomeric type II chaperonin (Chapple et al., 2001). Due to the similarity between BBS6, BBS10 and BBS12, it seems feasible to speculate that these three BBS proteins could form a larger, multi-subunit complex, possibly in conjunction with other, yet unknown proteins. Therefore, studies addressing whether BBS6 alone or together with BBS10 and BBS12 forms a novel chaperonin complex could provide further insights into disease pathogenesis and lead to the identification of new chaperonin client proteins, which might represent causative BBS genes.

### 3.3 Prefoldin 5

Prefoldin (PFDN, also termed GimC complex, genes involved in microtubule biogenesis complex) is a heterohexameric chaperone that cooperates in the folding of nascent protein chains with cytosolic chaperonin CCT. The interaction of PFDN with non-native protein chains protects them from aggregation and degradation until their transfer to CCT, where the final protein folding steps occur (Vainberg et al., 1998). Therefore, PFDN can be considered to function as a co-chaperonin, even though its role seems to be different from so-called classical co-chaperonins, such as GroES, which acts as a cap for the GroEL cylinder. PFDN is a 90 kDa complex, comprising two alpha and four beta subunits, which form a jellyfish-like structure (Fig. 1). PFDN binds the exposed hydrophobic residues of unfolded proteins via a so-called hydrophobic groove at the distal end of one of the mobile beta subunits. Following β subunit–client interaction the α subunits change their conformations and positions. This leads to an adjustment of the width of the central PFDN cavity depending on the mass of the bound substrate (Ohtaki et al., 2007).

The major targets for PFDN binding are considered to be actin, as well as α and β tubulin. The binding of unfolded actin and PFDN results in a conformational change of the actin protein giving rise to a rod-like shaped protein, which is then recognized by CCT. It seems likely that the unfolded actin chain requires folding into a quasi-native conformation before an interaction with CCT can occur. In addition, PFDN does not release the pre-folded protein into the cytosol, but physically interacts with CCT, forming a PFDN:client:CCT complex (Martin-Benito et al., 2002; Zako et al., 2005).

Interestingly, introduction of a point mutation in the prefoldin 5 (*PFDN5*) gene was reported to cause abnormal photoreceptor outer segment development in a recessive mouse model of retinal dystrophy (NMF5a corresponding to *rd13*) (Lee et al., 2006; Abstract presented at the Association for Research and Vision 2006, Poster no. 2296). This mutation, which was introduced by ENU mutagenesis, leads to the amino acid substitution L110R in PFDN5. In the mutant mice outer and inner segment cell thickness was reduced compared to WT littermate controls. In addition, rhodopsin and rom1, a protein important for regulation of morphogenesis and stabilization of the outer segment, were mislocalized in the mutant mice. These findings suggest that transport via the connecting cilium to the outer segment might be impaired or poorly developed in the PFD mutant mice. The cytoskeleton of the connecting cilium consists mainly of microtubules and actin fibres, which are crucial for transport of molecules from the outer segment and morphogenesis of the outer segment disk membranes, respectively. Since microtubules and actin are the main client proteins of PFDN binding it seems likely that mutations in *PFDN5* impair the correct folding of vital components of the connecting cilium, compromising its integrity. However, this hypothesis does not explain why the observed phenotype of the PFDN5 mutation is mainly retinal. Further characterization of this mouse model may reveal novel chaperone roles for PFDN in the retina, possibly by interacting with retina specific proteins or other molecules relevant for retinal function.

### 3.4 AIPL1

Leber congenital amaurosis (LCA) is a genetically heterogeneous, inherited retinopathy characterized by rapid degeneration of rod and cone photoreceptors shortly after birth (Sohocki et al., 2000). Blindness normally occurs within the first year of life, making this the most severe form of inherited retinal dystrophy. LCA has been shown to be caused by mutations in the photoreceptor and pineal-specific aryl hydrocarbon receptor interacting protein-like 1 (*AIPL1*) (Sohocki et al., 2000), and investigations are currently aimed at dissecting the normal functions of this protein.

AIPL1 is expressed in both rod and cone photoreceptors of the developing retina (van der Spuy et al., 2003) but is restricted to rods in the adult retina (van der Spuy et al., 2002). The reason for this developmental switch is unclear but indicates an important role of AIPL1 in mature rod cell function, in addition to a role in functional development of the human retina. AIPL1 was named due to its homology (49% identity) with the Aryl hydrocarbon receptor (AhR)-interacting protein (AIP), also known as the human X-associated protein 2 (XAP2) or AhR-activated 9 (AhR9) (Kuzhandaivelu et al., 1996; Carver and Bradfield, 1997; Ma and Whitlock, 1997). AIP is a co-chaperone similar to the immunophilins (FKBP51/52), which interact with the Hsp90 chaperone machinery through their tetratricopeptide (TPR) motif to promote the functional maturation of their respective client proteins, the AhR and the steroid hormone receptors. AIPL1 has 3 TPR motifs (van der Spuy, 2006). The conservation of the TPR domain in AIPL1 suggests that it may function as a retina-specific co-chaperone involved in the regulation of cognate clients in an analogous manner to AIP. Indeed, it has recently been shown that AIPL1 interacts with both Hsp90 and Hsp70, and that disease-causing mutations within the TPR domains diminish these associations (Hidalgo de Quintana et al., 2008).

**Fig. 4:**
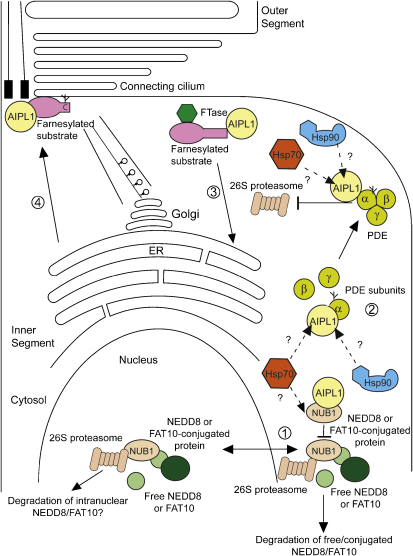
Potential roles of AIPL1 in photoreceptors. Schematic representation showing potential roles of AIPL1 within photoreceptors. AIPL1 is able to modulate the nuclear localization of NUB1, which may affect the NUB1 NEDD8 and FAT10 ‘busting’ activity (1); AIPL1 interacts with and enhances stability of the PDE holoenzyme (2); AIPL1 may enhance transport and stability of farnesylated proteins to the ER (3) or other target membranes (4). AIPL1 is likely to utilize the Hsp70 and Hsp90 chaperone machinery to execute its cellular functions.

Several potential AIPL1 client proteins have been identified, through a combination of yeast two hybrid screens and analyses of animal models. One potential client is the widely expressed NEDD8 ultimate buster 1 (NUB1), which has been identified as an AIPL1-interacting protein (Akey et al., 2002). NUB1 is predominantly a nuclear protein involved in orchestrating the proteasomal degradation of the ubiquitin-like proteins NEDD8 and FAT10 and their cellular targets (Kito et al., 2001; van der Spuy et al., 2003; Hipp et al., 2004). It appears to function as an adaptor protein for the proteasome through associations mediated by its ubiquitin-like (UBL) and ubiquitin-associated (UBA) domains. Interestingly, AIPL1 was shown to modulate the nuclear translocation of NUB1 in a manner analogous to the action of AIP on the AhR (van der Spuy and Cheetham, 2004) (Fig. 4). In addition, AIPL1 acted like a chaperone to suppress the aggregation of NUB1 fragments into inclusion bodies. This effect was specific for NUB1, as it had no effect on inclusion formation of other aggregation-prone proteins, such as P23H rod opsin or a mutant polyglutamine expanded huntingtin fragment (van der Spuy and Cheetham, 2004). More recently, AIPL1 was shown to cooperate with Hsp70 in reducing the formation of NUB1 fragment inclusions (Hidalgo de Quintana et al., 2008). It will also be of interest to determine whether AIPL1 can affect the NUB1-mediated proteasomal degradation of free and conjugated NEDD8 and FAT10 (Fig. 4). Both of these small ubiquitin-like proteins have been implicated in cell cycle regulation, suggesting that AIPL1 may regulate photoreceptor growth and development through modulating NUB1 activity, although animal models of AIPL1 LCA suggest that AIPL1 is not required for photoreceptor development and differentiation *per se* (Dyer et al., 2004; Ramamurthy et al., 2004; Liu et al., 2004).

AIPL1 has also been shown to interact with and enhance processing of the farnesylated proteins γ-transducin and the Hsp40 protein HDJ2 (Ramamurthy et al., 2003). Retinal lysates from AIPL1 knockout and hypomorphic mice, however, did not show global defects in farnesylation, but a specific reduction in rod cGMP phosphodiesterase (PDE), a farnesylated heterotrimeric protein complex that plays an essential role in visual phototransduction (Ramamurthy et al., 2004; Liu et al., 2004). Importantly, all three subunits of the PDE holoenzyme (PDEα, β and γ) were down-regulated at the protein but not mRNA level prior to disease progression, suggesting AIPL1 may play a role in the synthesis, assembly and/or stabilization of the PDE protein complex. In this case, PDE may represent a retina-specific client of AIPL1, whereby AIPL1 interacts specifically with PDEα and enhances its farnesylation, protecting it from proteasomal degradation. Given that AIPL1 also interacts with Hsp90 and Hsp70 (Hidalgo de Quintana et al., 2008), the resulting chaperone heterocomplex may be involved in the facilitation of PDE assembly or quality control of the subunits (Fig. 4). In any case, the list of retina-specific client proteins that mediate AIPL1 chaperone function remains to be completed, and future studies are likely to reveal interesting new insights into the retina-specific chaperone activities of AIPL1.

## 4 Conclusions

The retina offers a unique opportunity to study molecular chaperone function in a highly specialized and accessible part of the CNS. Several retinal dystrophy genes have mutations that make the proteins misfold and aggregation prone. The availability of good *in vitro* and *in vivo* models will allow the dissection of the mechanisms of cell death associated with these misfolding events and testing of therapies based on manipulating molecular chaperones. The importance of chaperones to retinal function is reiterated by the multiplicity of diseases caused by loss of specific chaperones. Studying these chaperones in more detail may enhance our understanding, not only of photoreceptor function, but also the biology of chaperone networks.

## Figures and Tables

**Table 1:** Examples of retinal disease genes that have protein misfolding as part of their pathogenesis

Disease	Disease gene	Function/putative function	Loss or gain of function	References
Autosomal dominant retinitis pigmentosa (ADRP)	Rhodopsin	Rod photoreceptor light receptor	Gain of function and dominant negative	Illing et al. (2002), Saliba et al. (2002), Rajan and Kopito (2005)
	Inosine monophosphate dehydrogenase type I (IMPDH1) (RP10)	IMPDH1 catalyzes the rate-limiting step of guanine nucleotide synthesis	Gain of function	Aherne et al. (2004)
	Carbonic anhydrase IV (RP17)	Catalyzation of the reversible hydration of carbon dioxide for buffering extracellular pH levels	Loss of function or gain of function	Bonapace et al. (2004), Rebello et al. (2004), Yang et al. (2005)
Bardet–Biedl Syndrome (BBS) and MMKS	MKKS/BBS6	Centrosomal protein, required for cilia/basal body function	Loss of function	Hirayama et al. (2007)
Best vitelliform macular dystrophy	Bestrophin 1 (best 1)	Regulation of voltage gated Ca(2+) channels	Loss of function	Kunzelmann et al. (2007), Burgess et al. (2008)
Doyne honeycomb macular dystrophy	EGF-containing fibulin-like extracellular matrix protein 1	Extracellular matrix protein of unknown function	Loss of function	Marmorstein et al. (2002)
Leber congenital amaurosis (LCA) and early onset retinal dystrophy (EORD)	RPE65	Retinoid isomerase	Loss of function	Hamel et al. (1993), Chen et al. (2006), Takahashi et al. (2006)
	AIPL1	Retina-specific co-chaperone	Loss of function	Ramamurthy et al. (2003), van der Spuy and Cheetham (2004)
Spinocerebellar ataxia 7	SCA7	Component of TFTC and STAGA transcription factor complexes	Gain of function	Bowman et al. (2005)
Stargardt disease	Elongase of very long chain fatty acids-4 (ELOVL4)	Elongation of very long chain fatty acids	Gain of function and dominant negative	Karan et al. (2004), Grayson and Molday (2005)
	ATP-binding cassette (ABC) transporter A4 (ABCA4)	Transports *all-trans* retinal and/or derivatives across outer segment discs dependent manner	Loss of function	Wiszniewski et al. (2005)
X-linked progressive retinal atrophy (XLPRA)	RPGR (retinitis pigmentosa GTPase regulator)	Function unknown, protein localizes to centrioles, ciliary axonemes and microtubular transport complexes	Loss of function	Zhang et al. (2002)
X-linked retinoschisis	Retinoschisin (RS1)	Cell adhesion protein	Loss of function	Wang et al. (2002), Wu and Molday (2003)

**Table 2:** Retinal dystrophies associated with mutations in chaperones or co-chaperones

Disease	Disease gene	Phenotype	Chaperone system	Function/putative function	References
Bardet–Biedl Syndrome (BBS)	McKusick–Kaufman Syndrome (MKKS)/BBS6	Retinal degeneration	Type II chaperonin homology	Centrosomal protein, possibly involved in cilia/basal body function	Stone et al. (2000), Katsanis et al. (2000), Kim et al. (2005), Ross et al. (2005), Hirayama et al. (2007)
		Obesity			
		Post-axial polydactyly			
	BBS10	Retinal degeneration	Type II chaperonin homology	Centrosomal protein, possibly involved in cilia/basal body function or protein–protein interactions	Stoetzel et al. (2006)
		Obesity			
		Cognitive impairment			
		Genito-urinary tract malformations			
	BBS12	Retinal degeneration	Type II chaperonin homology	Centrosomal protein, possibly involved in cilia/basal body function or protein–protein interactions	Stoetzel et al. (2007)
		Obesity			
		Cognitive impairment			
		Genito-urinary tract malformations			
Leber congenital amaurosis (LCA)	Aryl hydrocarbon receptor interacting protein-like 1 (AIPL1)	Blindness at birth	TPR protein, Hsp70/Hsp90 co-chaperone	Modulation of NUB1 nuclear translocation; interaction with and facilitation of protein farnesylation; post-translational synthesis, biogenesis or assembly of phosphodiesterase (PDE) subunits	Sohoki et al. (2000), Ramamurthy et al. (2003, 2004), van der Spuy and Cheetham (2004, 2006), Liu et al. (2004), Hidalgo de Quintana et al. (2008)
		No detectable ERG			
		Retinal dysfunction and degeneration			
NFM5a (*rd13*) mutant mouse	Prefoldin (PFDN) 5	Photoreceptor degeneration, abnormal outer segment development	PFD chaperone system	Actin and tubulin folding	Lee et al. (2006)
X-linked retinitis pigmentosa (XLRP)	RP2	Retinal degeneration	Homology to tubulin specific chaperone C (TBCC)	Plasma membrane localization, involvement in tubulin biogenesis	Schwahn et al. (1998), Chapple et al. (2000), Grayson et al. (2002)
